# Don’t forget to safeguard the lungs during DCD multi-organ retrieval! Early *in situ* oxygenated preservation matters

**DOI:** 10.3389/ti.2026.17507

**Published:** 2026-10-01

**Authors:** Carlos Jordá Aragón, Alilis Fontana Bellorín

**Affiliations:** Department of Thoracic Surgery and Lung Transplant, Hospital Universitari i Politècnic La Fe, Valencia, Spain

**Keywords:** A-NRP, donation after circulatory death (DCD), donation after circulatory determination of death (DCDD), lung transplant, TA-NRP

We are writing regarding the recently published international consensus statement by the European Society for Organ Transplantation (ESOT) establishing standards for controlled donation after circulatory determination of death (cDCDD) in adults [1], as well as the accompanying report detailing the results of the ESOT Bucharest consensus conference on the use of normothermic regional perfusion (NRP) in this donor population [2].

While those documents mark a major milestone in standardizing cDCDD protocols across Europe, there is increasing concern within the thoracic transplant community that the unique physiological requirements of lung preservation have received comparatively limited attention, particularly in contrast to the detailed recommendations addressing abdominal organ preservation (a-NRP) and, more recently, thoraco-abdominal preservation (TA-NRP) driven by heart retrieval [1, 2]. In both scenarios, abdominal and cardiac graft management is strictly protocolized. However, in the preservation of the lung allograft, the special physiological mechanism of the lung, the so-called “pulmonary privilege”, appears to be frequently overlooked [3–5]. Following the mandatory hands-off period, current clinical practices typically alternate between regional perfusion and immediate static cold storage, thereby delaying lung reinflation until sternotomy and pulmonary artery cannulation [2].

Framing lung preservation strategy exclusively around the a-NRP versus TA-NRP debate is conceptually flawed from a pulmonary perspective. Unlike other solid organs, the lung parenchyma does not depend on continuous blood perfusion to sustain aerobic cellular metabolism [6]; direct alveolar oxygen diffusion into the interstitial space and vascular endothelium is sufficient to prevent ATP depletion and ischemic cell collapse [3–5]. Consequently, the only effective mechanism to preserve cellular metabolism during the immediate post-mortem period is alveolar oxygenation, achieved either through protective mechanical ventilation or continuous positive airway pressure (CPAP) with a high (0.5) fraction of inspired oxygen ( ${\text{FiO}}_{2}$ ) [3–6].

Crucially, the timing of re-ventilation must be meticulously balanced with bioethical safeguards. The duration of the mandatory post-mortem observation or “no-touch” period varies internationally between countries from 3 to 20 min, with a global average of 5 min [4]. From an ethical and physiological standpoint, a minimum of 5 min should be respected following cardiac arrest to avoid auto-resuscitation of the heart. Initiating mechanical ventilation prematurely within this 5-min window must be strictly avoided, as the hypoxic cardiac arrest might otherwise get reversed in the donor, or residual cerebral oxygenation could be compromised, violating the dead donor rule [7].

Certainly, immediately after this mandatory standoff period has been fully respected and death has been legally declared, initiating mechanical ventilation or CPAP represents “minute zero” of lung preservation. This early alveolar recruitment must not be deferred until chest opening in the operating room [3]. Pathophysiological principles strongly support this strategy [6], international donor procurement guidelines advise it [3, 4], and current ethical and legal regulations fully permit it as a post-mortem preservation maneuver [7].

The lung cannot remain an overlooked organ in global DCD standards [1, 2]. In cDCDD cases managed with abdominal NRP (a-NRP), the overall preservation environment should be recognized as a dual-temperature preservation [8] (abdominal normothermia combined with thoracic hypothermic/ischemic oxygenated preservation) [3, 4]. Rather than relying solely on hypothermia to slow down metabolic rates, early alveolar oxygen supply delivers true oxygenated ischemia, which preserves pneumocyte viability and allograft quality over extended periods [3, 4, 6].

Moreover, technical standardization in cDCDD managed with TA-NRP must extend beyond ventilatory parameters to address pulmonary hydrostatic safety. Reanimating the donor heart inherently exposes the pulmonary vasculature to active perfusion. The ESOT Bucharest Consensus explicitly acknowledges the threat of lung congestion and cardiac distension during TA-NRP, its guidelines rely on indirect right-sided decompression by optimizing venous return to the ECMO circuit via a centrally inserted dual-stage venous cannula [2]. This approach, however, fails to prevent post-capillary congestion caused by left ventricular stunning or transient contractility failure. Therefore, Van Raemdonck et al. argue that direct and immediate venting of the left atrium, by inserting a venting catheter via the interatrial groove at the initiation of TA-NRP, is required to prevent hydrostatic pulmonary edema. They argue that upon restoring antegrade perfusion, blood pools retrogradely into the left heart while the ventricular apparatus is still arrested or stunned, causing devastating post-capillary damage if an active outflow tract is not provided [9].

This direct approach, however, introduces significant surgical complexity and suffers from a profound lack of standardization. The AATS 2024 Expert Consensus [3] confirms that the clinical utilization of lungs after TA-NRP remains highly controversial and data are limited [10, 11], pointing directly to “inconsistent venting practices” as the primary cause of hydrostatic edema and variable graft outcomes. To date, there is no high-quality clinical evidence confirming that this *in situ* surgical maneuver increases the rate of valid donors. On the contrary, its technical difficulty in an unstable donor, combined with the absence of a uniform protocol, frequently deters transplant teams from accepting these grafts altogether out of fear of acute hydrostatic edema.

At present, how to reliably protect the pulmonary vascular bed during TA-NRP remains a poorly standardized frontier, as current guidelines fail to offer a clear, consensus-driven protective roadmap. Within this gap, inconsistent venting practices represent a critical technical failure, and a lapse in procedural leadership that acts as a powerful deterrent for transplant teams, unnecessarily limiting lung graft utilization. Furthermore, we must address the strategic allocation of high-cost healthcare resources: while *Ex Vivo* Lung Perfusion (EVLP) is an indispensable, vital “safety net” to evaluate and rescue borderline grafts [3], particularly those with suspected hydrostatic edema, it must never serve as a routine, high-cost bypass for suboptimal *in situ* preservation. Shifting the burden of graft protection from the donor’s bedside to *ex situ* platforms is both logistically and financially unsustainable; clinical priority must remain focused on rigorous, proactive *in situ* donor care.

Ultimately, while current consensus guidelines remain heavily focused on complex hepatic or cardiac preservation protocols, they frequently overlook simple, cost-effective, and highly viable maneuvers to safeguard the lung graft [1, 2]. While the transplant community debates highly invasive and poorly standardized *in situ* surgical venting procedures to mitigate hydrostatic injury, the simplest protective strategy of all - early *in situ* alveolar oxygenation via ventilation or CPAP immediately following the mandatory 5-min no-touch period - remains unrecognized as a standardized frontline maneuver. Shifting the immense burden of pulmonary protection from the donor’s bedside to high-cost *ex situ* platforms like EVLP is logistically and economically unsustainable [3]. A paradigm shift is urgently required: transitioning from static cold storage toward early *in situ* oxygenated preservation, as successfully demonstrated in uncontrolled donation after circulatory death (uDCD) [12]. The lung must no longer be the forgotten organ in regional perfusion strategies and cDCDD consensus guidelines.

## Data Availability

The original contributions presented in the study are included in the article/supplementary material, further inquiries can be directed to the corresponding author.
